# The impact of group membership on punishment versus partner rejection

**DOI:** 10.1038/s41598-024-69206-9

**Published:** 2024-09-27

**Authors:** Trystan Loustau, Jacob Glassman, Justin W. Martin, Liane Young, Katherine McAuliffe

**Affiliations:** Department of Psychology and Neuroscience, Boston College, 275 Beacon St, Chestnut Hill, MA 02467 USA

**Keywords:** Psychology, Human behaviour

## Abstract

People often display ingroup bias in punishment, punishing outgroup members more harshly than ingroup members. However, the impact of group membership may be less pronounced when people are choosing whether to stop interacting with someone (i.e., partner rejection). In two studies (N = 1667), we investigate the impact of group membership on both response types. Participants were assigned to groups based on a “minimal” groups paradigm (Study 1) or their self-reported political positions (Study 2) and played an incentivized economic game with other players. In this game, participants (Responders) responded to other players (Deciders). In the Punishment condition, participants could decrease the Decider’s bonus pay. In the Partner Rejection condition, participants could reject future interactions with the Decider. Participants played once with an ingroup member and once with an outgroup member. To control for the effects of intent and outcome, scenarios also differed based on the Decider’s Intent (selfish versus fair) and the Outcome (equal versus unequal distribution of resources). Participants punished outgroup members more than ingroup members, however group membership did not influence decisions to reject partners. These results highlight partner rejection as a boundary condition for the impact of group on responses to transgressions.

## Introduction

Group membership influences a variety of behaviors, including cooperation, conflict, empathy, trust, and many others^1–4^. Of recent interest is the tendency for group membership to influence how people respond when others transgress against them (e.g., when they harm them or others, violate a norm, or otherwise behave antisocially). Much of this work has focused on one type of response: punishment—the imposition of a cost on a transgressor^5–8^. Past research has found that outgroup members are often punished more harshly than ingroup members by third-party observers^9–12^. However, in other cases, third-party observers may instead punish ingroup members more harshly than outgroup members^13^, such as when there is high certainty of guilt^14^. While work on third-party punishment points to an important role for group membership, these data do not speak to how individuals react when they themselves are the victims of the transgression. This distinction is crucial because the dynamics of personal involvement in an offense may lead to different patterns of punitive behavior compared to third-party punishment.

Relatively less work has examined ingroup bias in an interpersonal context (i.e., second-party punishment). However, what work exists has yielded mixed findings, with some studies showing that people punish outgroup members more harshly^15^, other studies showing that people punish ingroup members more harshly^16^, and yet other studies showing no clear effect of group membership on punishment in an interpersonal context^17–19^. To help clarify these effects, we examine the impact of group membership on second-party punishment decisions in the present work.

Importantly, punishment is not the only way people can respond to others’ transgressions. They may also respond with partner rejection, refusing to interact with the transgressor any further^20,21^. Similar to punishment, partner rejection is an important and frequently employed method for navigating social relationships and social conflict^22^. Partner rejection can be observed in many areas of life, such as in the termination or rejection of friendships, romantic relationships, employment opportunities, roommate and housing situations, or club memberships. Thus, it is important to consider the extent to which partner rejection is influenced by group membership. Examining the sensitivity of partner rejection to group membership is also critical to clarifying the boundary conditions for the impact of group membership status on responses to transgressions. In the present work, we investigate the influence of group membership on both punishment and partner rejection in response to interpersonal transgressions.

### Distinguishing punishment and partner rejection

Traditional accounts of interpersonal punishment, that is, punishment inflicted upon a transgressor by the victim of a transgression, have defined it as the imposition of a cost on a transgressor to change a behavior^23^. These costs can vary; they may be monetary (e.g., pay cut or fine), physical (e.g., corporal punishment), or psychological and emotional (e.g., reputational damage). Modern accounts of punishment suggest that punishment serves a communicative function, demonstrating that people infer that punishment will be effective if it communicates social and moral norms even when it poses no cost to the transgressor^24^. This account is supported by prior work showing that punishment signals and enforces moral values and social rules^5,25–29^. Given the communicative role of punishment, it makes sense that punishment is sensitive to group membership.

Punishing outgroup members more harshly than ingroup members communicates lower tolerance for mistreatment by outgroup members than ingroup members^30^. As demonstrated by prior work, the impact of group membership on punishment is flexible and responsive to the communicative goals of the punisher. For instance, ingroup members are often punished more harshly than outgroup members in the context of group deviance, communicating lower tolerance for such deviance by ingroup members than outgroup members^16^.

Most research relevant to the function of partner rejection focuses on *partner choice* broadly, which includes both partner *selection* (i.e., choosing to interact with someone) and partner *rejection* (i.e., choosing not to interact with someone or not to continue interacting with someone). Indeed, some work using the term *partner choice* measures only one of the two components—partner selection or partner rejection^31^. For clarity, we discuss relevant literature on partner choice, but primarily use the term partner rejection, as that was the focus of the present work.

Within the traditional accounts of punishment outlined above, partner rejection might be classified as a form of punishment that imposes costs through the severance of a social tie (e.g., emotional pain, loss of resources, and social connection). Yet, in contrast to punishment, partner choice serves as a tool for navigating the “biological market”^32^, a conceptual framework where individuals compete to form beneficial cooperative relationships based on the relative benefits they can offer each other^20,33,34^. Partner choice decisions are sensitive to market dynamics, as partner selectors and rejectors aim to maximize their personal benefits, creating pressure for potential partners to make themselves look appealing to partner selectors and for current partners to behave cooperatively^20,21^. As evidence of this, participants tend to behave more cooperatively when participants can choose or reject their partners in economic games^35,36^.

Given the market function of partner choice decisions for maximizing one’s own benefits, partner choice may only be sensitive to group membership to the extent that group membership provides information regarding the impact that a particular partnership (new or continued) will have on personal well-being. For example, people may be more likely to choose interaction partners from ingroups that are trusted to be more cooperative than outgroups that are not. However, when group membership does not provide much information about a potential partner’s generosity and fairness, it may not be factored into partner choice decisions. Indeed, one study found that participants’ choices for a partner before an economic game were not sensitive to whether they belong to the same ethnic or gender group but were sensitive to how reliable they perceived them to be based on a photograph^37^. Similarly, people’s decisions to reject a partner who has mistreated them may be insensitive to the partner’s group membership, given that group membership is unrelated to the mistreatment.

This account of partner choice, that emphasizes individual well-being, is consistent with prior work showing that second-party partner choice decisions are more strongly influenced by the intentions of interaction partners (i.e., to be fair or selfish) than the outcome (e.g., unequal or equal distribution of resources) of the interactions^38,39^. Seeking to maximize their individual well-being, people may reject partners who intended to be selfish even if the outcome was equal in order to protect themselves from future mistreatment. By contrast, the same work shows that decisions to punish an interaction partner are relatively equally influenced by their intentions and the outcome. This balanced consideration of intentions and outcomes supports the role of punishment in both addressing the immediate injustice and signaling norms and expectations for future interactions.

Both partner rejection and punishment represent ways of responding to an individual transgressor. In the present work, we investigate the impact of group membership on partner rejection and punishment in response to individual transgressions when group membership is not inherently tied to the transgressions. We examine the impact of group membership on the likelihood of partner rejection and the likelihood of punishment separately, as well as directly compare the strength of impact of group membership on these two types of responses. Building on previous work highlighting that punishment and partner rejection are differentially sensitive to intent and outcome, we designed studies that allow us to incorporate those differences while investigating the impact of group membership.

### Current studies

In the present work, we explore the following question: “How does group membership impact second-party decisions to punish or reject a partner?” Across two studies, we examine participants’ punishment and partner rejection decisions in an online economic game adopted from past work. We manipulate group membership using a multi-pronged approach, through a “minimal” groups paradigm (Study 1) and based on a self-reported political opinion (Study 2). In addition, in order to account for differences in punishment and partner rejection that may be due to the perceived intent of the partner and the outcome of the interaction, intent and outcome varied within-subjects. All methods were carried out in accordance with relevant guidelines and regulations. Informed consent was obtained from all participants and all procedures for both studies were approved by the Boston College Institutional Review Board.

## Study 1

### Method

#### Participants

American participants (n = 802) were recruited via Amazon Mechanical Turk and completed an online survey in exchange for a small payment (≤ $2.00) with an opportunity to earn a bonus payment. We preregistered a target minimum sample size of n = 300 per condition. We excluded data from participants who failed comprehension questions or attention checks, who were not native English speakers, or whose mean reaction time was less than three standard deviations below the overall mean. These criteria yielded a final sample of 659 participants (17.8% excluded; final sample 48% male, 51% female, < 0.5% other, < 0.5% preferred not to answer). The percentage excluded was similar across both response type conditions (Punishment: 14.9%; Partner Rejection: 11.3%).

Post-hoc sensitivity analyses were conducted using the simr package^40^ in R Studio to determine the minimum effect sizes that we were powered to detect in a linear mixed effects model and a logistic regression with the current sample. Using simr sensitivity analyses with 1000 simulations, we found that the linear mixed effects model was 81.00% (95% CI 71.93, 88.16) powered to detect a three-way interaction of GroupXIntentXOutcome with an unstandardized beta of 0.13, and that the logistic regression model was 80.60% (95% CI 78.01, 83.01) powered to detect a three-way interaction of GroupXIntentXOutcome with an unstandardized beta of 1.74 (OR 5.70).

#### Procedures

First, participants were assigned to one of two minimal groups. Then, they interacted with other participants in an asynchronous online economic game modeled on past work^39,41^. Participants were randomly assigned to play one of two versions of the game based on response type: Punishment or Partner Rejection.

##### Group assignment

In Study 1, we employed a variant of the “minimal” groups paradigm^2,42^ to assign group membership. After providing consent, participants were told they would perform a task that would determine which team they were on for the economic game. Participants were presented with a 3 × 3 word search and told to enter the first three-letter word they saw into a free response box. Participants were then told which team they belonged to based on the word they entered, TEAM CAT or TEAM OWL, and selected one of five potential cat or owl avatars to represent them throughout the task. To promote group identification, we informed participants that “people on the same team tend to share many personality and cognitive traits.” This paradigm assigned participants to groups about which they know almost nothing, allowing us to investigate the influence of the abstract group membership on punishment and partner rejection.

##### Economic game

Participants were then given instructions for the economic game. This game involved two roles: The Decider and the Responder. First, participants played as the Responder. On each trial, the Decider was required to choose one of two different methods for allocating $1 between themselves and the Responder: Option A and Option B. Option A resulted in a 2/3 chance of giving the full $1 to the Decider and a 1/3 chance of giving $0.50 to the Decider and $0.50 to the Responder. Option B resulted in a 2/3 chance of giving $0.50 to the Decider and $0.50 to the Responder and a 1/3 chance of giving the full $1 to the Decider. Thus, these options were designed such that Option A was more likely to yield an unequal outcome than Option B, but both options could have yielded an equal or unequal outcome. In this way, the Decider’s intent was dissociable from the outcome, resulting in four potential interactions: selfish intent and unequal outcome, selfish intent and equal outcome, fair intent and unequal outcome, and fair intent and equal outcome.

Participants played the game twice as a Responder: once with an ingroup member and once with an outgroup member in a randomized order. For each trial, participants were presented with all four possible combinations of the Decider’s choice and outcome (Table 1) and were asked to commit to a response for each option.

**Table 1:** Four conditions (Studies 1–2).

		Decider’s Intent	
		Option A (selfish)	Option B (fair)
Outcome	Equal	Selfish Intent | Equal Outcome	Fair Intent | Equal Outcome
	Unequal	Selfish Intent | Unequal Outcome	Fair Intent | Unequal Outcome

Participants were randomly assigned to one of two response conditions: Punishment or Partner Rejection. In the Punishment Condition, participants had the opportunity to add or subtract up to $0.30 in $0.10 increments from the Decider’s payoff. In both conditions, the decision to change Deciders or to punish was always costless. In the Partner Rejection Condition, participants were given a binary choice to either play another round with the same Decider or to play another round with a new Decider (i.e., reject the current decider). If the participant decided to reject their current Decider, the new Decider would always be from the same group as their current Decider (e.g., if they rejected a Decider from TEAM CAT, the Decider would be replaced with another Decider from TEAM CAT). In this way, neither the partner rejection nor punishment response could change the group which a participant’s Decider was from.

In both conditions, participants responded to all four potential outcomes. This method achieved two things: (1) we gained four times the amount of data than we would have using “hot” decisions, and (2) we could ask for participants’ decisions without informing them of the Decider’s decision or outcome on that round, so that the decisions between rounds were not influenced by the decisions or outcomes of previous rounds.

##### Rating group identification

After playing the game with both an ingroup and outgroup player, participants completed identification ratings for their ingroup and outgroup in a randomized order. We assessed these ratings after participants’ economic game participation to avoid the potential demand effects of assessing identification immediately following group assignment. Participants rated their agreement with four statements drawn from prior work (e.g., “I like TEAM [Owl/Cat]”^43^) on a 100-pt slider scale from “Strongly disagree” to “Strongly agree”. These ratings had high reliability (Ingroup: Cronbach’s alpha = 0.92, 95% CI 0.90–0.92; Outgroup: Cronbach’s alpha = 0.82, 95% CI 0.80–0.84), so the ratings for each group were averaged for each participant.

##### Additional measures

After playing as Responder, participants were told they would play this game two more times in the role of Decider. This provided us with Deciders to match with Responders. Procedures and findings for participants’ decisions as Deciders are reported in the Supplementary Materials. At the end of the study, participants completed eight attention check items, completed an optional demographics questionnaire, and were debriefed.

##### Analysis approach

All analyses were conducted in RStudio. Since responses in the Punishment Condition were on an interval scale (-$0.30 to $0.30 by $0.10) and responses in the Partner Rejection Condition were binary (reject or keep), it was not possible to compare these conditions in the same regression model. Therefore, we used linear mixed effects regression to analyze data from the Punishment Condition and logistic regression to analyze data from the Partner Rejection Condition. Group Membership (Ingroup versus Outgroup), Decider’s Intent (Fair versus Selfish), and Outcome (Equal versus Unequal) were entered as predictors in the full models. We also conducted exploratory analyses using a classification approach to determine the proportion of participants who were sensitive to intentions, outcomes, and group membership in each condition. Results for these exploratory analyses for each study are reported in the Supplementary Materials.

## Results and discussion

### Manipulation check

Participants identified more strongly with their ingroup than their outgroup in both response type conditions, *t*(658) = 24.37,* p* < 0.001, *d* = 1.34 (Table 2). We found no difference between conditions in identification with ingroup members, *t*(654.63) = 1.41,* p* = 0.16, *d* = 0.11, or outgroup members, *t*(649.55) = 0.69,* p* = 0.49, *d* = 0.05. Thus, our group manipulation was successful in both response type conditions.

**Table 2:** Mean group identification ratings (Studies 1 and 2).

Study	Mean (SEM)		
	Ingroup	Neutral	Outgroup
Study 1	62.48 (0.91)	NA	40.97 (0.71)
Study 2	72.44 (0.87)	40.17 (0.87)	29.58 (0.96)

	Comparison	Test	
Study 1	Ingroup vs. Outgroup	*t*(658) = 24.37,* p* < 0.001, *d* = 1.34	
Study 2	Ingroup vs. Outgroup	*t*(599) = 29.85,* p* < 0.001, *d* = 1.72	
Study 2	Ingroup vs. Neutral	*t*(599) = 27.64,* p* < 0.001, *d* = 1.60	

### Punishment condition

We conducted a series of linear-mixed effects regression with the lme4 package^44^ to analyze data from the Punishment Condition. The models included a random intercept for each participant as well as random slopes for group membership, intent, and outcome. We used a model comparison approach by evaluating the importance of predictors using Likelihood Ratio Tests. First, we compared the full model with the three-way interaction term (Group MembershipXIntentXOutcome) to the same model without the three-way interaction term. The three-way interaction term did not improve model fit, (LRT *X*^2^ (1) = 0.01, *p* = 0.932). Next, we compared the model with all two-way interaction terms to the same model without the two-way interaction terms. We found that the two-way interaction terms improved model fit (LRT *X*^2^ (3) = 12.53, *p* = 0.006). The two-way interaction model is reported in Table 3.

**Table 3:** Linear mixed effects regression on punishment (Study 1).

Effect	*B*(SE)	t	*p*	95% CI
Intent	− 0.41 (0.05)	− 8.58	< 0.001	(− 0.50, − 0.31)
Outcome	− 0.73 (0.05)	− 15.98	< 0.001	(− 0.82, − 0.64)
Group Membership	0.06 (0.02)	3.81	< 0.001	(0.03, 0.09)
IntentXOutcome	− 0.09 (0.03)	− 3.48	< 0.001	(− 0.14, − 0.04)
IntentXGroup Membership	− 0.02 (0.03)	− 0.65	0.515	(− 0.07, 0.03)
OutcomeXGroup Membership	− 0.004 (0.03)	− 0.14	0.888	(− 0.05, 0.05)

**Figure 1:**
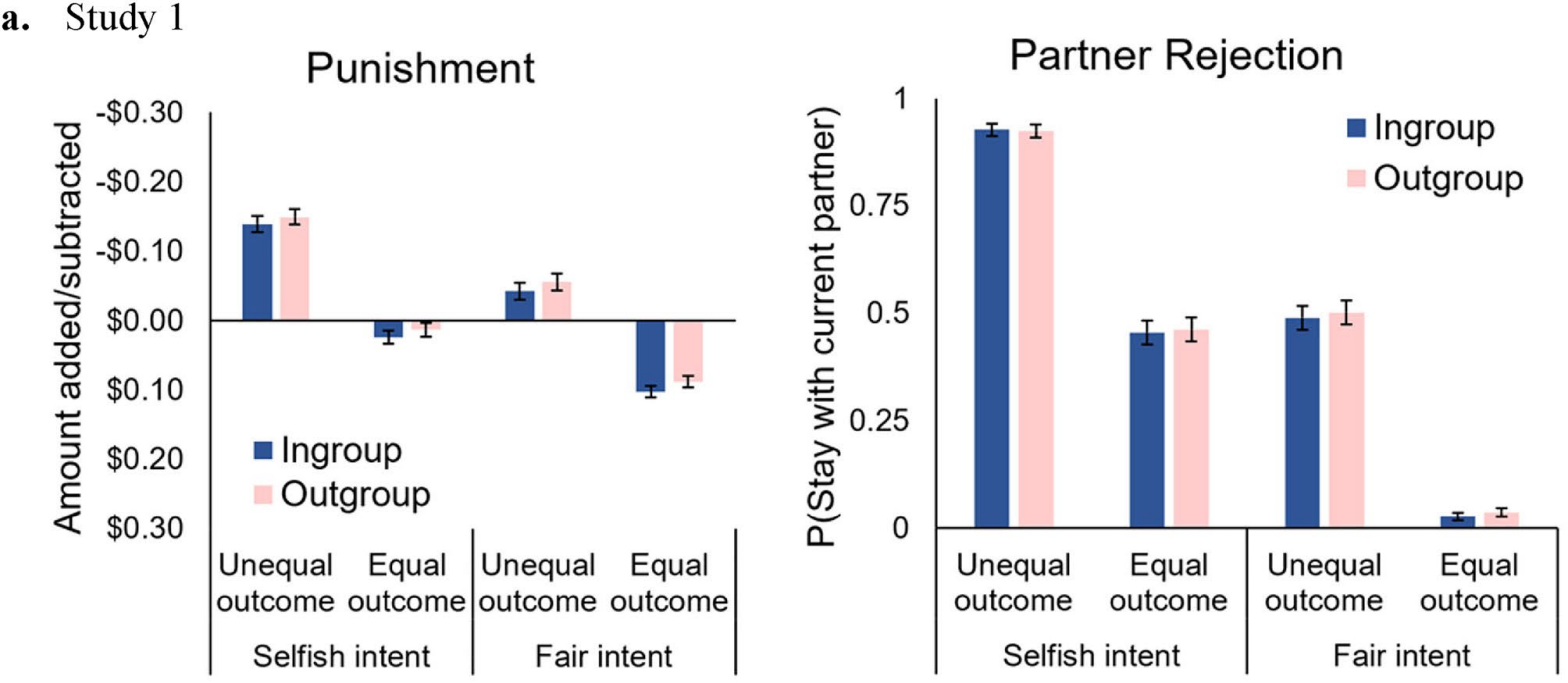
Mean responses in the punishment and partner rejection conditions in study 1. Plotted are participants’ mean responses across the Intention, Outcome, and Group membership conditions for the Punishment (Panel **A**) and Partner Choice (Panel **B**) conditions. Error bars are SEM.

We found that there was a significant main effect of Group Membership (Table 4) such that participants punished less (i.e., subtracted less from the Decider’s payoff) when the Decider was an ingroup member than when they were an outgroup member. There was a significant main effect of Intent such that participants punished less when the Decider had a Fair intent than when they had a Selfish intent. Additionally, there was a significant main effect of Outcome such that participants punished less when the Outcome was Equal than when it was Unequal. There was a significant interaction between Intent and Outcome (Table 5) such that participants punished fair Deciders less than selfish Deciders, especially when the outcome was unfair than when the outcome was fair. The main punishment scores for each condition, grouped by group membership status, are displayed in Fig. 1, panel a. Since punishment responses between $0.00 and $0.30 may not be interpreted as punishment, we also conducted a supplemental analysis excluding these responses. The results, available in the supplementary materials, replicate these findings.

**Table 4:** Mean engagement in punishment or partner rejection (Studies 1–2).

Study	Response type	Mean or proportion (SEM)		
		Ingroup	Neutral	Outgroup
1	Punishment (Mean)	− 0.01 (0.01)	NA	− 0.03 (0.01)
	Partner Rejection (Proportion)	0.53 (0.01)	NA	0.52 (0.01)
2	Punishment (Mean)	0.29 (0.01)	0.33 (0.01)	0.42 (0.01)
	Partner Rejection (Mean)	0.44 (0.01)	0.45 (0.01)	0.47 (0.01)

**Table 5:** Impact of intent and outcome on punishment and partner rejection (Studies 1–2).

Study	Intent	Outcome	Response type	
			Punishment	Partner Rejection
1	Fair	Equal	0.10 (0.01)	0.97 (0.01)
	Fair	Unequal	− 0.05 (0.01)	0.50 (0.02)
	Selfish	Equal	0.02 (0.01)	0.54 (0.02)
	Selfish	Unequal	− 0.14 (0.01)	0.07 (0.01)
2	Fair	Equal	0.10 (0.01)	0.04 (0.01)
	Fair	Unequal	0.48 (0.02)	0.45 (0.02)
	Selfish	Equal	0.18 (0.01)	0.44 (0.02)
	Selfish	Unequal	0.63 (0.02)	0.87 (0.01)

Punishment scores represent Means and SEMs. Partner Rejection scores represent Proportions and SEMs in Study 1 and Means and SEMs in Study 2.

### Partner rejection condition

First, we conducted a logistic mixed effects regression to analyze data in the Partner Rejection Condition, with a random intercept entered for each participant. However, results indicated that there was zero variance for this random intercept, so we reverted to standard logistic regression. The results of the logistic regression model are reported in Table 6. Results showed that, in contrast to the Punishment Condition, there was no significant main effect of group membership. There was a significant main effect of Intent such that participants were more likely to reject the Decider when they had a selfish intent than when they had a fair intent. There was also a significant main effect of Outcome such that participants were more likely to reject the Decider when there was an unequal outcome than when there was an equal outcome. Additionally, there was a significant interaction between Intent and Outcome such that participants were more likely to reject Deciders with a selfish intent than those with a fair intent, especially when the outcome was unequal. The effects of Intent and Outcome were consistent with the findings from the Punishment Condition. The main partner rejection scores for each condition grouped by group membership status are displayed in Fig. 1, panel b.

- Study 1
- Study 2

**Table 6:** Logistic regression on partner rejection (Study 1).

Effect	*b*(SE)	z	*p*	95% CI
Intercept	0.27 (0.07)	3.737	< 0.001	(0.13, 0.42)
Intentions	− 2.89 (0.15)	− 19.89	< 0.001	(− 3.19, − 2.62)
Outcomes	− 3.04 (0.15)	20.901	< 0.001	(− 3.34, − 2.76)
Group Membership	0.08 (0.15)	0.562	0.574	(− 0.20, 0.37)
IntentionsXOutcomes	0.72 (0.29)	2.472	0.013	(0.16, 1.31)
Group Membership XIntent	− 0.18 (0.29)	− 0.629	0.530	(− 0.76, 0.38)
Group Membership X Outcome	− 0.16 (0.29)	− 0.545	0.586	(− 0.74, 0.41)
IntentionsXOutcomeXGroup Membership	0.18 (0.58)	0.309	0.758	(− 0.96, 1.34)

## Study 2

In Study 1, we found that participants punished outgroup members more harshly than ingroup members but did not reject outgroup members more than ingroup members. However, since directly comparing the effect of group membership on punishment and partner rejection was not possible, it may not be possible to conclude that the impact of group membership was different between the two response types. It is possible that a small effect size was detected by the large sample size in the punishment condition. Therefore, in Study 2, we measured both punishment and partner rejection decisions on a binary scale, allowing us to analyze data from both conditions within the same regression model.

While the minimal group paradigm we utilized in Study 1 allowed us to examine the impacts of abstract group membership, the lack of sensitivity to group boundaries in the Partner Rejection Condition may reflect the relatively weak nature of the minimal group manipulation. To address this concern in Study 2, we assigned participants to groups based on their position on a consequential political issue (i.e., abortion). Assigning groups in this way invokes more salient boundaries which are frequently relevant in daily life. Utilizing these two approaches to group assignment allowed us to combine the strengths of both to obtain a better understanding of how group membership impacts responses to interpersonal transgressions, in line with prior work^45^.

In Study 2, we also included a third type of group member with which participants interacted: a neutral individual (who did not feel strongly about the political issue), which allowed us to examine whether the ingroup bias we observed in the Punishment Condition in Study 1 is more reflective of ingroup love (i.e., preferential treatment of an ingroup member compared to non-ingroup members) or outgroup derogation (i.e., preferential treatment of non-outgroup members compared to outgroup members).

In sum, Study 2 built upon the results of Study 1 by (1) directly comparing punishment and partner rejection by measuring them both on the same scale, (2) examining the impact of group membership on punishment and partner rejection in a real-world group context, and (3) comparing the influence of ingroup and outgroup membership to a control (neutral group membership).

### Method

#### Participants

American participants (n = 865) were recruited via Amazon Mechanical Turk and completed an online survey in exchange for a small payment (≤ $2.00) with an opportunity to earn a bonus payment. We preregistered a target minimum sample size of n = 300 per condition. We excluded data from participants based on the same exclusion criteria used in Study 1 in addition to data from those who reported no position on the political issue used to assign participants to a group. These criteria yielded a final sample of 600 participants (23.2% excluded; final sample 47% male, 52% female, < 0.5% other, < 0.5% preferred not to answer). The percentage excluded was very similar across our response type conditions (Punishment: 22.7%; Partner Choice: 23.8%).

A post-hoc sensitivity analysis was conducted using the simr package^40^ in R Studio to determine the minimum effect size (Odd’s Ratio) that this study was powered to detect in a two-way interaction, as our main analysis examined the interaction between group membership and response type (punishment versus partner choice). This study had 0.80 power to detect an effect size of at least 0.62 or 1.60 with a 5% error rate.

#### Procedures

In Study 2, group membership was determined based on opinions about a political issue and participants played a modified version of the economic game used in Study 1.

##### Group assignment

After providing consent, participants were asked a series of standard demographic questions, and about their stance on abortion. Participants could identify as “Pro-Choice”, “Pro-Life” or “No position” and rated how strongly they supported this position on a Likert scale from 1 = “Not strongly at all” and 7 = “Very strongly”. Participants and the other players they interacted with were subsequently identified with an image corresponding to their choice.

##### Economic game

Participants played the same economic game from Study 1, with a few modifications. First, participants played the game three times instead of twice, once with a player from each group (Pro-life, Pro-choice, Neutral). The order of these interactions was counterbalanced across participants. Second, unlike the continuous measure of punishment in Study 1, there were only two response options available in the Punishment Condition. Since the most frequently chosen punishment options in Study 1 were to not punish (41% of responses) and maximum punishment (removing $0.30; 27% of responses), we used these as the punishment options available in Study 2.

##### Rating group identification

After playing the game with all three players, participants completed the same four-item measure of group identification used in Study 1 for each of the three groups. These ratings had high reliability (Ingroup: 0.91, 95% CI 0.89–0.91; Outgroup: 0.92, 95% CI 0.91–0.93; Neutral: 0.89, 95% CI 0.88–0.90) and so the ratings for each group were averaged for each participant.

##### Additional measures

As in Study 1, participants also played the game three times as the Decider. In an exploratory manner, the 7-item Cognitive Reflection Test (CRT^46^) was administered after the game. Results for the Decider role and the CRT are reported in the Supplementary Materials. At the end of the study, participants completed eight attention check items and were debriefed.

##### Analysis approach

As specified in our pre-registration, we used mixed-effects logistic regression to examine the impact of response type and group membership on response. Selecting punishment or partner rejection was coded as 1, and selecting no punishment or partner rejection was coded as 0. Response Type (Punishment versus Partner Rejection), Group Membership (Ingroup versus Outgroup versus Neutral), Intent (Selfish versus Fair), and Outcome (Unequal versus Equal) were entered as fixed effects, as well as all possible interactions between these variables. We also included a random intercept for participants. As in Study 1, we used a model comparison approach to evaluate our data using Likelihood Ratio Tests.

## Results and discussion

### Manipulation check

We found that participants in both conditions identified with their ingroup significantly more than their outgroup or neutral individuals (Table 2). Identification with each group did not differ between response type conditions. Thus, our group manipulation successfully led participants to identify more with their own group compared to the two other groups.

### Results

First, we compared the full model with the four-way interaction term (ResponseTypeXGroup MembershipXIntentXOutcome) to the same model without the four-way interaction term. The four-way interaction term did not improve model fit (LRT *X*^2^ (2) = 0.38, *p* = 0.829). Next, we compared the model with all three-way interaction terms to the same model without the three-way interaction terms. We found that the three-way interaction terms significantly improved model fit (LRT *X*^2^ (7) = 14.37, *p* = 0.045). The results of the three-way interaction model are reported in Table 7.

**Table 7:** Mixed effects logistic regression analysis (Study 2).

Effect	B (SE)	OR	SE	z	*p*	95% CI
Intercept	− 1.23 (0.10)***	0.29	0.10	− 12.33	< 0.001	(0.24, 0.35)
Group Membership: Neutral	0.26 (0.10)*	1.29	0.10	2.54	0.011	(1.06, 1.58)
Group membership: Outgroup	0.78 (0.10)***	2.18	0.10	8.10	< 0.001	(1.84, 2.64)
Intent	2.09 (0.16)***	8.11	0.16	13.45	< 0.001	(6.23, 11.02)
Outcome	3.31 (0.16)***	27.49	0.16	20.94	< 0.001	(20.49, 39.25)
Response Type	− 1.08 (0.19)***	0.34	0.19	− 5.76	< 0.001	(0.24, 0.49)
Intent X Group member: Netural	− 0.05 (0.21)	0.95	0.21	− 0.25	0.801	(0.65 1.36)
Intent X Group member: Outgroup	− 0.22 (0.20)	0.80	0.20	− 1.12	0.262	(0.55 1.14)
Outcome X Group member: Netural	− 0.08 (0.21)	0.92	0.21	− 0.39	0.693	(0.63, 1.39)
Outcome X Group member: Outgroup	− 0.66 (0.19)***	0.52	0.19	− 3.39	< 0.001	(0.35, 0.75)
Intent X Outcome	− 0.85 (0.31)**	0.43	0.31	− 2.76	0.006	(0.24, 0.75)
Response type X Group member: Netural	0.22 (0.18)	1.25	0.18	1.22	0.221	(0.89, 1.79)
Response type X Group member: Outgroup	0.75 (0.18)***	2.13	0.18	4.30	< 0.001	(1.48, 3.03)
Response type X Intent	− 1.94 (0.28)***	0.14	0.28	− 6.98	< 0.001	(0.08, 0.24)
Response type X Outcome	0.13 (0.29)	1.14	0.29	0.45	0.652	(0.64, 2.05)
Intent X Outcome X Group member: Netural	0.37 (0.42)	1.45	0.42	0.89	0.372	(0.64, 3.19)
Intent X Outcome X Group member: Outgroup	0.98 (0.39)*	2.66	0.39	2.52	0.012	(1.27, 5.58)
Intent X Response type X Group member: Netural	− 0.20 (0.37)	0.82	0.37	− 0.54	0.590	(0.40, 1.62)
Intent X Response type X Group member: Outgroup	0.02 (0.36)	1.02	0.36	0.05	0.959	(0.50 2.05)
Outcome X Response type X Group member: Neutral	− 0.12 (0.39)	0.88	0.39	− 0.32	0.750	(0.43, 1.92)
Outcome X Response type X Group member: Outgroup	− 0.43 (0.37)	0.65	0.37	− 1.15	0.250	(0.31 1.36)
Intent X Response type X Outcome	0.60 (0.32)	1.83	0.32	1.89	0.058	(0.99, 3.89)

***p < 0.001, **p < 0.01, *p < 0.05.

There was a significant interaction between Group Membership and Response Type such that participants were more likely to punish/reject outgroup members compared to ingroup members and neutral group members, especially in the Punishment Condition compared to the Partner Rejection Condition (Table 1). Indeed, follow-up analyses on the data from the Partner Rejection Condition revealed that the three-way interaction term (Group MembershipXIntentXOutcome) did not improve model fit (LRT *X*^2^ (2) = 4.04, *p* = 0.13) and, while two-way interaction terms improved model fit (LRT *X*^2^ (5) = 14.45, *p* = 0.01), we found no interactions with group membership (all OR < 1.11 and > 0.72, *p* > 0.20).

**Figure 2:**
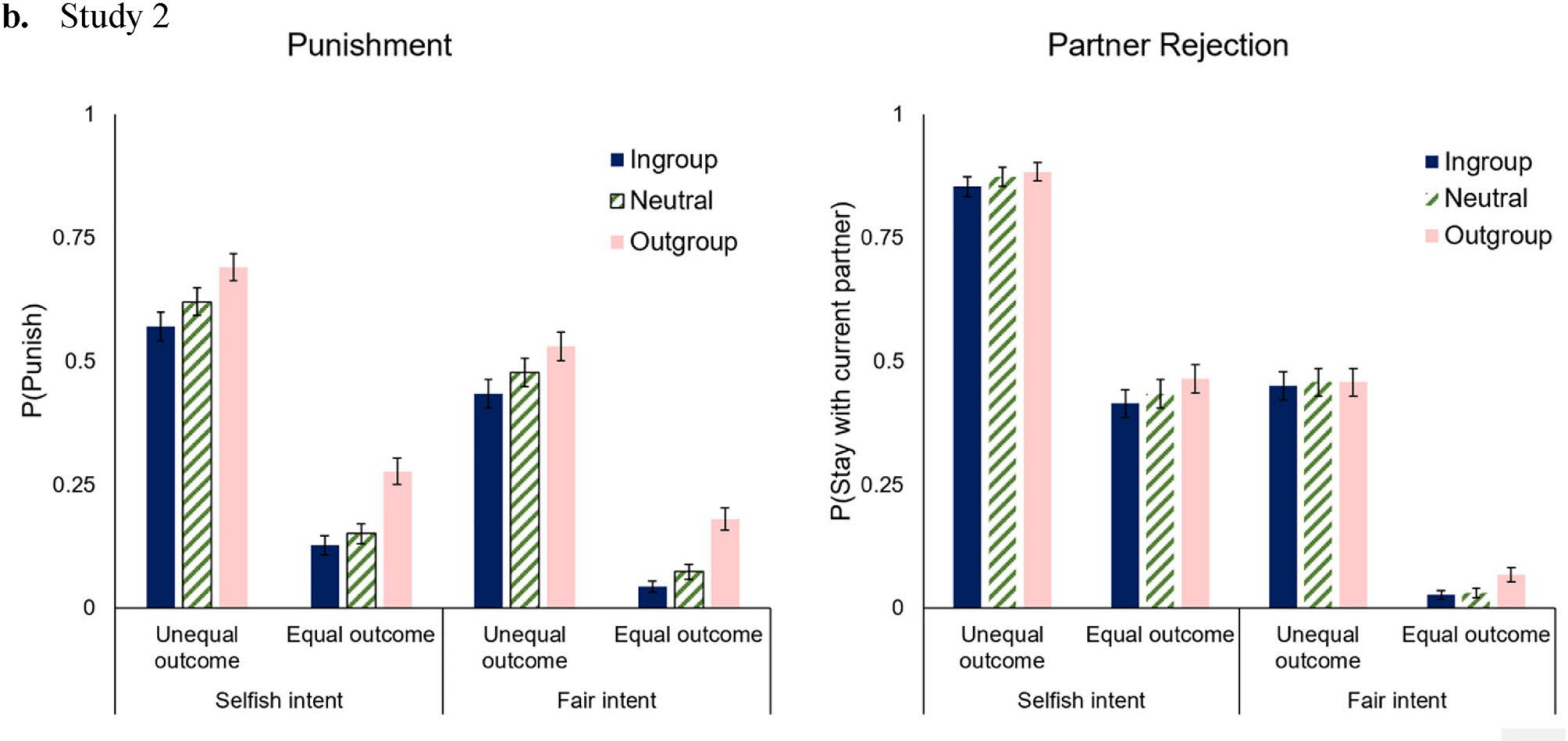
Mean responses in the Punishment and Partner Rejection conditions in Study 2. Plotted are participants’ mean responses across the Intention, Outcome, and Group membership conditions for the Punishment (Panel **A**) and Partner Choice (Panel **B**) conditions. Error bars are SEM.

We found a main effect of Group Membership such that participants were more likely to punish/reject outgroup members than ingroup members and neutral group members, but no more likely to punish/reject neutral members compared to ingroup members. In contrast to Study 1, there was a significant interaction between Group Membership and Outcome such that participants were more likely to punish/reject outgroup members compared to ingroup members, especially when there was an equal outcome compared to when there was an unequal outcome. The main punishment and partner rejection scores for each condition grouped by group membership status are displayed in Fig. 2.

Additionally, we found a significant three-way interaction between Group Membership, Intent, and Outcome such that the tendency to punish/reject outgroup members more than ingroup members when the outcome was equal compared to when it was unequal was slightly greater when the intent was fair than when it was selfish (Table 8).

**Table 8:** Mean engagement in punishment or partner rejection (Study 2).

Intent	Outcome	Mean (SEM)		
		Ingroup	Neutral	Outgroup
Fair	Equal	0.04 (0.01)	0.05 (0.01)	0.12 (0.01)
Fair	Unequal	0.44 (0.02)	0.47 (0.02)	0.49 (0.02)
Selfish	Equal	0.27 (0.02)	0.29 (0.02)	0.37 (0.02)
Selfish	Unequal	0.71 (0.02)	0.75 (0.02)	0.79 (0.02)

Replicating the results of Study 1, we found a significant main effect of Intent such that participants were more likely to punish/reject when Deciders had a selfish intent than when they had a fair intent. Additionally, there was a significant main effect of Outcome such that participants were more likely to punish/reject when there was an unequal outcome than when there was an equal outcome. Once again, there was a significant interaction between Intent and Outcome such that participants were more likely to punish/reject selfish Deciders than fair Deciders, especially when the outcome was unfair than when the outcome was fair (Table 5).

We found a significant main effect of Response Type such that participants rejected partners more often than they punished partners. There was a significant interaction between Response Type and Intent such that the tendency to punish/reject Deciders with a selfish (versus fair) intent was stronger for partner rejection compared to punishment.

## General discussion

Across two studies, we investigated the impact of group membership on two ways of responding to others’ transgressions: punishment and partner rejection. Participants in both studies interacted with other players in an online economic game for real stakes. We assigned participants to groups using a “minimal” groups paradigm (Study 1) and a consequential political position (Study 2). In line with prior work^15^, we find that people punish outgroup members more often and more harshly than ingroup members, especially when the partner’s intent was selfish and the outcome was unequal. Extending this work, we find that, in contrast, people are not sensitive to group membership when deciding whether to reject their partner. This effect is robust across both methods of assigning group membership.

Why might punishment be sensitive to group membership in a way that partner rejection is not? One potential explanation centers on the functional differences between punishment and partner rejection. While punishment often involve a coordinated response to help maintain the well-being of the group or define group boundaries, partner rejection may involve unilateral decisions which have more immediate and personal consequences for individual well-being. Thus, when deciding to reject a partner or not, people may place more weight on factors that have a more direct impact on their well-being, such as the partner’s intent and the outcome of the interaction, than on group membership, which does not directly impact their well-being. Conversely, punishment may serve to maintain and strengthen group boundaries by imposing more costs on outgroup members than ingroup members. By punishing outgroup members more harshly than ingroup members, people may act in accordance with perceived group norms regarding differential treatment of ingroup versus outgroup members. Ingroup bias in punishment decisions may therefore also serve a group-strengthening function, strengthening participant’s attachment to their ingroup and their detachment from the outgroup. Our findings align with broader work showing that moral responses are most sensitive to categorical distinctions when norm enforcement is more important^47^. Thus, the influence of categorical distinctions such as ingroup-outgroup status may be amplified in contexts such as punishment where enforcing social norms and maintaining group cohesion are key concerns.

There are two additional possibilities for why we observed a weaker impact of group membership on partner rejection versus punishment. First, participants in the partner rejection condition were told that rejected partners would be replaced with another partner from the same group as the rejected partner. Although this was done to ensure that both punishment and partner rejection decisions could not change the group status of the partner, this could have mitigated the impact of group membership on the decision to reject by limiting the implications of rejection for signaling consequences for a partner’s whole group. Future work could examine the impact of group rejection on partner choice when rejection decisions replace partners with one from another group. Second, given that ingroup favoritism tends to be strongest in economic games with single versus multiple trials^48^, telling participants in the partner rejection condition that a rejection decision would result in a new partner may have mitigated the effect of group membership by increasing the expectation of continued interaction with the same group. Future work could examine how the impact of group membership on punishment versus partner choice is influenced by the expectation of future interactions.

Given the costs of punishing alone, punishment is often coordinated between multiple members of a group^49,50^. Views of punishment as a coordinated act suggest that such coordination and collective-decision making are likely to be influenced by the prevailing norms and values of the group, increasing the relevance of ingroup-outgroup status distinctions. Additionally, punishment may be less likely to be influenced by the intentions of the actor, which are not necessarily commonly known. Indeed, in the current work, we found that intentions had a stronger impact on partner rejection than on punishment, supporting the idea that punishment may evoke collective or group-based thinking, which is less sensitive to individual motivations. These findings are broadly consistent with prior work suggesting that partner choice is more sensitive to intentions, while punishment is more sensitive to outcomes^39^. However, given that we did not observe an interaction between Response Type and Outcome in Study 2, more work is needed to explore how and why the impact of intentions and outcomes on punishment and partner rejection may vary. Future work should continue to examine the psychological mechanisms responsible for the difference between punishment and partner rejection in sensitivity to group membership.

Building on prior work on third-party punishment^10^, we also found that people were more likely to punish outgroup members compared to neutral individuals. Although participants were more likely to punish neutral individuals than ingroup members, this difference was not significant. These results suggest that ingroup bias in the context of punishment is driven more by outgroup derogation than ingroup love, consistent with other work on intergroup bias^51,52^. However, alternative interpretations of these data exist; for instance, participants may have been more likely to punish outgroup members due to greater perceived norm violations, reduced empathetic restraint, or fewer expected future interactions. Indeed, previous work has shown that outgroup derogation is limited^53,54^. It is also possible that the difference in punishment toward neutral individuals and ingroup members in our study may have been artificially reduced by the fact that in the equal outcome conditions, punishment rates were near floor for all types of group members. Indeed, prior work^11^ has found that neutral individuals are punished more than ingroup members. Given this, future work should continue to explore the robustness of differences in punishment between ingroup members and neutral individuals.

### Limitations

In the present work, we largely explored differences in punishment and partner rejection when they are costless for the responders. Since prior work has demonstrated a consistent influence of group membership on punishment both when afflicting punishment is costly or costless for responders^30,55–57^, we do not expect that our results for the Punishment Condition would have been different if punishment decisions were costly. However, it is possible that group membership may have a stronger impact on partner rejection when partner rejection decisions are costly. If partner rejection functions primarily to maintain individual well-being, partner rejection decisions may be more sensitive to costs that differentially impact well-being in the context of different social groups. For example, the social costs associated with rejecting a partner from one’s own group may damage one’s reputation with the ingroup, limiting their access to resources or important social networks. The perceived threats of these lost social ties may override the perceived immediate benefits to one’s well-being for rejecting a transgressive partner. On the contrary, rejecting a partner from the outgroup may not pose the same social cost, and thus, would not pose as much of a long-term threat to individual well-being. Thus, the perceived immediate personal benefits of partner rejection may be more persuasive for rejecting an outgroup member. Future work should investigate the influence of group membership on partner rejection decisions when these decisions are costly. Such work could obtain a costly and continuous measure of partner choice using a pay-to-avoid paradigm, by which participants indicate how much they are willing to pay to avoid being matched with a person.

This work is also limited in that we investigated the influence of group membership on only second-party punishment and partner rejection decisions (i.e., those made by the victims of a transgression). The dyadic nature of these interactions may explain why we observed a stronger influence of intent and outcome on punishment and partner rejection than group membership. Indeed, prior work shows that people are especially more likely to punish outgroup members than ingroup members when the punisher is a third-party observer^12,30^ and less so when the punisher is the victim^17^. It is unclear how the role of partner rejection decisions might change when made by third-party observers compared to second-party victims. Just as second-party victims seek to maximize their welfare by rejecting partners that have previously harmed them, third-party observers might reject partners that have previously harmed others to maximize their welfare. It is possible that partner rejection decisions made by third-party observers also serve as a form of social signaling, similar to punishment. Given that third-party observers who reject a partner do so based on a harm they observed, rather than a harm they experienced, their rejection may signal group expectations around fair treatment. Future work should clarify the impact of group membership on partner rejection decisions made by third-party observers.

Another limitation of the present work is that we conducted the economic games with samples of online participants, who may be less attentive than participants who participate in economic games in person. A lack of attentiveness could explain why, at an absolute level, intent had a weaker impact on punishment/partner rejection decisions in our studies than in past work using in-person samples of undergraduate participants^38,39^. Follow-up analyses revealed that the impact of intent was stronger among participants who got more comprehension questions correct on their first try, as well as among participants who scored higher on the Cognitive Reflection Test. However, we excluded participants who were unable to answer comprehension checks questions correctly, so this difference in attentiveness should not have impacted participants’ understanding of the task. Additionally, controlling for attentiveness and CRT performance in our analyses did not change the influence of group membership between conditions. Future work should clarify what factors impact the strength of the influence of intent on punishment and partner choice.

In the present work, we were primarily focused on assessing actual, incentivized behavior in scenarios where decisions have tangible consequences. However, we recognize that experimental games likely do not capture the full complexity of real-world interactions. In this work, we aimed to bridge this gap by examining behaviors in both minimal groups and real-world group contexts. Nonetheless, future work could benefit from integrating vignette studies to explore these dynamics in more varied settings.

## Conclusion

Group-based psychology pervades our social life. When interacting with another person produces an unequal outcome for oneself, or suggests that the other person was behaving selfishly, decisions to punish or reject the person are differentially influenced by the person’s group membership status. Across minimal and consequential manipulations of group membership, we find that people are more likely to punish outgroup members than ingroup members, but no more likely to reject them. These results shed light on the underlying functions of punishment and partner rejection and expand our understanding of the role that groups play in our social decision-making.

## Supplementary Information

Supplementary Information.

## Data Availability

Data and analysis scripts for all studies can be found at https://osf.io/cjs8q/. For both studies, we report all measures, manipulations and exclusions. The pre-registration for Study 1 can be found at http://aspredicted.org/blind.php?x=j92vh7. The pre-registration for Study 2 can be found at http://aspredicted.org/blind.php?x=56xa9a. All materials can be found in the Supplementary Materials. All procedures for both studies were approved by the Boston College Institutional Review Board.

## References

[1] Cikara, M. Causes and consequences of coalitional cognition. in *Advances in Experimental Social Psychology* **64**, 65–128 (Elsevier, 2021).

[2] Dunham, Y. Mere membership. *Trends Cogn. Sci.* **22**, 780–793. 10.1016/j.tics.2018.06.004 (2018). 30119749 10.1016/j.tics.2018.06.004

[3] McAuliffe, K. & Dunham, Y. Group bias in cooperative norm enforcement. *Phil. Trans. R. Soc. B* **371**, 20150073. 10.1098/rstb.2015.0073 (2016). 26644592 10.1098/rstb.2015.0073 PMC4685519

[4] Tajfel, H. & Turner, J. An integrative theory of intergroup conflict. *The Social Psychology of Intergroup Relations* (1979).

[5] Boyd, R. & Richerson, P. Punishment allows the evolution of cooperation (or anything else) in sizable groups. *Ethol. Sociobiol.* **195**, 171–195 (1992).

[6] Clutton-Brock, T. & Parker, G. Punishment in animal societies. *Nature* **373**, 209–216 (1995). 7816134 10.1038/373209a0

[7] Raihani, N. J. & Bshary, R. The reputation of punishers. *Trends Ecol. Evol.*10.1016/j.tree.2014.12.003 (2015). 10.1016/j.tree.2014.12.003 25577128

[8] Raihani, N. J. & Bshary, R. Punishment: One tool, many uses. *Evol. Hum. Sci.* **1**, e12. 10.1017/ehs.2019.12 (2019). 37588410 10.1017/ehs.2019.12 PMC10427336

[9] Baumgartner, T., Götte, L., Gügler, R. & Fehr, E. The mentalizing network orchestrates the impact of parochial altruism on social norm enforcement. *Hum. Brain Mapp.* **33**, 1452–1469. 10.1002/hbm.21298 (2012). 21574212 10.1002/hbm.21298 PMC6870290

[10] Schiller, B., Baumgartner, T. & Knoch, D. Intergroup bias in third-party punishment stems from both ingroup favoritism and outgroup discrimination. *Evol. Hum. Behav.* **35**, 169–175. 10.1016/j.evolhumbehav.2013.12.006 (2014).

[11] Jordan, J. J., Mcauliffe, K. & Warneken, F. Development of in-group favoritism in children’s third-party punishment of selfishness. *Proc. Natl. Acad. Sci. U.S.A.*10.1073/pnas.1402280111 (2014). 10.1073/pnas.1402280111 PMC4156744 25136086

[12] Yudkin, D. A., Rothmund, T., Twardawski, M., Thalla, N. & Van Bavel, J. J. Reflexive intergroup bias in third-party punishment. *J. Exp. Psychol. Gen.* **145**, 1448–1459. 10.1037/xge0000190 (2016). 27632379 10.1037/xge0000190

[13] Shinada, M., Yamagishi, T. & Ohmura, Y. False friends are worse than bitter enemies: “Altruistic” punishment of in-group members. *Evol. Hum. Behav.* **25**, 379–393. 10.1016/j.evolhumbehav.2004.08.001 (2004).

[14] Van Prooijen, J. W. Retributive reactions to suspected offenders: The importance of social categorizations and guilt probability. *Pers. Soc. Psychol. Bull.* **32**, 715–726. 10.1177/0146167205284964 (2006). 16648197 10.1177/0146167205284964

[15] Diekhof, E. K., Wittmer, S. & Reimers, L. Does competition really bring out the worst? Testosterone, social distance and inter-male competition shape parochial altruism in human males. *PLoS one* **9**, e98977. 10.1371/journal.pone.0098977 (2014). 25075516 10.1371/journal.pone.0098977 PMC4116333

[16] Mendoza, S. A., Lane, S. P. & Amodio, D. M. For members only: Ingroup punishment of fairness norm violations in the ultimatum game. *Soc. Psychol. Pers. Sci.* **5**, 662–670. 10.1177/1948550614527115 (2014).

[17] Kubota, J. T., Li, J., Bar-David, E., Banaji, M. R. & Phelps, E. A. The Price of Racial Bias. *Psychol. Sci.* **24**, 2498–2504. 10.1177/0956797613496435 (2013). 24121413 10.1177/0956797613496435 PMC4028070

[18] Chuah, S. H., Hoffmann, R., Jones, M. & Williams, G. Do cultures clash? Evidence from cross-national ultimatum game experiments. *J. Econ. Behav. Organ.* **64**, 35–48 (2007).

[19] McAuliffe, K. & Dunham, Y. Fairness overrides group bias in children’s second-party punishment. *J. Exp. Psychol. Gen.* **146**, 485–494. 10.1037/xge0000244 (2017). 28383989 10.1037/xge0000244

[20] Barclay, P. Strategies for cooperation in biological markets, especially for humans. *Evol. Hum. Behav.* **34**, 164–175. 10.1016/j.evolhumbehav.2013.02.002 (2013).

[21] Barclay, P. Biological markets and the effects of partner choice on cooperation and friendship. *Curr. Opin. Psychol.* **7**, 33–38. 10.1016/j.copsyc.2015.07.012 (2016).

[22] Bayer, J. B., Triệu, P., Ellison, N., Schoenebeck, S. Y. & Falk, E. B. Rejection sensitivity and interaction quality in everyday life. *J. Social Pers. Relation.* **38**, 3646–3668 (2021).

[23] Johnston, J. M. Punishment of human behavior. *Am. Psychol.* **27**, 1033 (1972). 4563919 10.1037/h0033887

[24] Sarin, A., Ho, M. K., Martin, J. W. & Cushman, F. A. Punishment is organized around principles of communicative inference. *Cognition* **208**, 104544 (2021). 33383397 10.1016/j.cognition.2020.104544

[25] Barclay, P. Reputational benefits for altruistic punishment. *Evol. Hum. Behav.* **27**, 325–344. 10.1016/j.evolhumbehav.2006.01.003 (2006).

[26] Jordan, J. J., Hoffman, M., Bloom, P. & Rand, D. G. Third-party punishment as a costly signal of trustworthiness. *Nature* **530**, 473–476. 10.1038/nature16981 (2016). 26911783 10.1038/nature16981

[27] Fehr, E. & Gächter, S. Altruistic punishment in humans. *Nature* **415**, 137–140. 10.1038/415137a (2002). 11805825 10.1038/415137a

[28] Henrich, J. *et al.* Markets, religion, community size, and the evolution of fairness and punishment. *Science* **327**, 1480–1484. 10.1126/science.1182238 (2010). 20299588 10.1126/science.1182238

[29] Yamagishi, T. The provision of a sanctioning system as a public good. *J. Pers. Soc. Psychol.* **51**, 110–116. 10.1037/0022-3514.51.1.110 (1986).

[30] Delton, A. W. & Krasnow, M. M. The psychology of deterrence explains why group membership matters for third-party punishment. *Evol. Hum. Behav.*10.1016/j.evolhumbehav.2017.07.003 (2017).

[31] Martin, J. W., Young, L., & McAuliffe, K. *The psychology of partner choice.* (2019). 10.31234/osf.io/weqhz

[32] Noë, R. & Hammerstein, P. Biological markets: Supply and demand determine the effect of partner choice in cooperation, mutualism and mating. *Behav. Ecol. Sociobiol.* **35**, 1–11 (1994).

[33] Hammerstein, P. & Noë, R. Biological trade and markets. *Philos. Trans. R. Soc. B Biol. Sci.* **371**, 20150101. 10.1098/rstb.2015.0101 (2016). 10.1098/rstb.2015.0101 PMC4760201 26729940

[34] André, J. B. & Baumard, N. The evolution of fairness in a biological market. *Evolution* **65**, 1447–1456. 10.1111/j.1558-5646.2011.01232.x (2011). 21521194 10.1111/j.1558-5646.2011.01232.x

[35] Bednarik, P., Fehl, K. & Semmann, D. Costs for switching partners reduce network dynamics but not cooperative behaviour. *Proc. R. Soc. B Biol. Sci.* **281**, 20141661 (2014). 10.1098/rspb.2014.1661 PMC4150333 25122233

[36] Wu, J., Balliet, D. & Van Lange, P. A. Gossip versus punishment: The efficiency of reputation to promote and maintain cooperation. *Sci. Rep.rts* **6**, 23919 (2016). 10.1038/srep23919 PMC4819221 27039896

[37] Aksoy, B., Eckel, C. C. & Wilson, R. K. Can I rely on you?. *Games* **9**, 81 (2018).

[38] Liddell, T. M. & Kruschke, J. K. Ostracism and fines in a public goods game with accidental contributions: The importance of punishment type. *Judgm. Decis. Mak.* **9**, 523–547 (2014).

[39] Martin, J. W. & Cushman, F. To punish or to leave: Distinct cognitive processes underlie partner control and partner choice behaviors. *PloS one* **10**, e0125193 (2015). 25915550 10.1371/journal.pone.0125193 PMC4411127

[40] Green, P. & MacLeod, C. J. SIMR: An R package for power analysis of generalized linear mixed models by simulation. *Methods Ecol. Evol.* **7**, 493–498 (2016).

[41] Cushman, F., Dreber, A., Wang, Y. & Costa, J. Accidental outcomes guide punishment in a “trembling hand” game. *PloS one* **4**, e6699 (2009). 19707578 10.1371/journal.pone.0006699 PMC2726629

[42] Tajfel, H., Billig, M. G., Bundy, R. P. & Flament, C. Social categorization and intergroup behaviour. *Eur. J. Social Psychol.* **1**, 149–178 (1971).

[43] Leach, C. W. *et al.* Group-level self-definition and self-investment: A hierarchical (multicomponent) model of in-group identification. *J. Pers. Social Psychol.* **95**, 144 (2008). 10.1037/0022-3514.95.1.144 18605857

[44] Bates, D., Maechler, M., Bolker, B., Walker, S., Christensen, R. H. B., Singmann, H., .Green, P. Package ‘lme4’. (2009). http://lme4.r-forge.r-project.org.

[45] Goette, L., Huffman, D. & Meier, S. The impact of social ties on group interactions: Evidence from minimal groups and randomly assigned real groups. *Am. Econ. J. Microecon.* **4**, 101–115 (2012).

[46] Toplak, M. E., West, R. F. & Stanovich, K. E. Assessing miserly information processing: An expansion of the Cognitive Reflection Test. *Think. Reason.* **20**, 147–168 (2014).

[47] Yoeli, E., Burum, B., Dalkiran, N. A., Nowak, M., & Hoffman, M. The emergence of categorical norms. [Preprint] (2022). 10.21203/rs.3.rs-2050019/v1

[48] Imada, H., Romano, A. & Mifune, N. Dynamic indirect reciprocity: When is indirect reciprocity bounded by group membership?. *Evol. Hum. Behav.* **44**, 373–383. 10.1016/j.evolhumbehav.2023.05.002 (2023).

[49] Molleman, L., Kölle, F., Starmer, C. & Gächter, S. People prefer coordinated punishment in cooperative interactions. *Nat. Hum. Behav.* **3**, 1145–1153. 10.1038/s41562-019-0707-2 (2019). 31477909 10.1038/s41562-019-0707-2

[50] Boyd, R., Gintis, H. & Bowles, S. Coordinated punishment of defectors sustains cooperation and can proliferate when rare. *Science* **328**, 617–620. 10.1126/science.1183665 (2010). 20431013 10.1126/science.1183665

[51] Halevy, N., Bornstein, G. & Sagiv, L. “In-group love” and “out-group hate” as motives for individual participation in intergroup conflict: A new game paradigm. *Psychol. Sci.* **19**, 405–411 (2008). 18399895 10.1111/j.1467-9280.2008.02100.x

[52] Hewstone, M., Rubin, M. & Willis, H. Intergroup bias. *Annu. Rev. Psychol.* **53**(1), 575–604 (2002). 11752497 10.1146/annurev.psych.53.100901.135109

[53] Mifune, N., Simunovic, D. & Yamagishi, T. Intergroup biases in fear-induced aggression. *Front. Psychol.* **8**, 49. 10.3389/fpsyg.2017.00049 (2017). 28174553 10.3389/fpsyg.2017.00049 PMC5258755

[54] Yamagishi, T. & Mifune, N. Parochial altruism: Does it explain modern human group psychology?. *Curr. Opin. Psychol.* **7**, 39–43. 10.1016/j.copsyc.2015.07.015 (2016).

[55] Bernhard, H., Fischbacher, U. & Fehr, E. Parochial altruism in humans. *Nature* **442**, 912–915 (2006). 16929297 10.1038/nature04981

[56] Stagnaro, M. N., Dunham, Y. & Rand, D. G. Profit versus prejudice: Harnessing self-interest to reduce in-group bias. *Soc. Psychol. Pers. Sci.* **9**, 50–58 (2018).

[57] Valenzuela, A. & Srivastava, J. Role of information asymmetry and situational salience in reducing intergroup bias: The case of ultimatum games. *Pers. Social Psychol. Bull.* **38**, 1671–1683 (2012). 10.1177/0146167212458327 22956295

