# Supplementary material for: The impact of group membership on punishment versus partner rejection
Source: Sci Rep. 2024 Sep 27;14:22238. doi: 10.1038/s41598-024-69206-9 (PMC11436655; doi:10.1038/s41598-024-69206-9)
Supplement: Supplementary file 1 — Supplementary Information. [file 41598_2024_69206_MOESM1_ESM.docx]

**Supplementary**

**Odds Ratios for Study 1 Partner Rejection Results**

**Supplementary Table 1**

*Logistic Regression on Partner Rejection (Study 1)*

| **Effect** | **OR(SE)** | **z** | **p** | **95% CI** |
| --- | --- | --- | --- | --- |
| Intercept  Intentions | 1.31 (0.07)  0.06 (0.15) | 3.737  -19.89 | <.001  <.001 | (1.14, 1.52)  (0.04, 0.07) |
| Outcomes | 0.05 (0.15) | 20.901 | <.001 | (0.04, 0.06) |
| Group Membership | 1.09 (0.15) | 0.562 | .574 | (0.8, 1.45) |
| IntentionsXOutcomes  Group Membership XIntent  Group Membership X Outcome  IntentionsXOutcomeXGroup Membership | 2.05 (0.29)  0.83 (0.29)  0.85 (0.29)  1.20 (0.58) | 2.472  -0.629  -0.545  .309 | .013  .530  .586  .758 | (1.18, 3.69)  (0.47, 1.47)  (0.48, 1.51)  (0.38, 3.80) |

**Results for Punishment in Study 1 Using Only the Negative Side of the Response Scale**

**Supplementary Table 2**

*Linear Mixed Effects Regression on Punishment (Study 1)*

| **Effect** | ***B*(SE)** | **t** | **p** | **95% CI** |
| --- | --- | --- | --- | --- |
| Intent | -0.27(0.04) | -6.47 | <.001 | (-0.35, -0.18) |
| Outcome | -0.94(0.06) | -16.86 | <.001 | (-1.06, -0.84) |
| Group Membership | 0.03(0.02) | 2.41 | .017 | (0.01, 0.07) |
| IntentXOutcome | -0.12(0.03) | -4.12 | <.001 | (-0.18, -0.06) |
| IntentXGroup Membership | -0.01(0.03) | -0.21 | .828 | (-0.03, 0.08) |
| OutcomeXGroup Membership | 0.03(0.03) | 0.96 | .337 | (-0.05, 0.05) |

**Analysis Approach for Study 1**

Including random slopes generated significant convergence issues. To address convergence issues, we ran standard regression models run with Firth bias correction and found that all significant fixed effects were supported or a greater number of significant effects, indicating that the random intercept models did not inflate type 1 error rate.

**Order Effects in Study 1**

We found a complicated pattern based upon the order in which participants encountered ingroup and outgroup Deciders. Participants weighted outcomes more heavily for the first Decider they encountered, staying if this decider caused a fair outcome and switching if they caused an unfair outcome. Importantly, we continued to find no overall influence of group membership, nor any interaction between group membership and intentions or outcomes.

**Exploratory Measures in Study 2**

**Playing as Decider.** Participants then played the game three more times, now playing as the Decider. Participants were provided with the same set of instructions but now described from the Decider’s perspective. Participants again answered a series of 7 comprehension questions, presented only once and not used in screening data quality. Participants played the game once with each type of player, with order counterbalanced across participants.

**CRT, attention check questions and debrief.** Participants next completed the 7-item CRT (Pennycook & Rand, 2020). Then they completed six questions designed to assess the degree to which the other player’s unfair choice and the unfair outcome were perceived as negative. For each type of player, participants were asked “When the partner who identified as [type] chose the unfair option (Option A), how negative did this make you feel?” and “When the partner who identified as [type] received the entire $1, how negative did this make you feel?”, with both questions asked on 7-point scales anchored at 1 = “Not at all negative” and 7 = “Extremely negative”. Finally, participants completed the same set of attention check questions as in Study 1 and were debriefed.

**Exploratory Analyses in Study 2**

*Firth Bias Correction*

Inspection of the main mixed-effects logistical regression model in Study 2 and the distribution of our response variable indicated that some cells of our design had very few responses. For instance, when paired with an Ingroup Decider, in the case where the Decider had a fair intent and produced a fair outcome, only 8/300 responses (0.3%) were to reject the Decider, and 13/300 responses (0.4%) were to engage in punishment. Similarly, when paired with a Neutral Decider, in the same case, only 9/300 responses (0.3%) were to reject the Decider and 22/300 responses (0.7%) were to engage in punishment. Typical procedures to address convergence issues were not able to overcome this issue of rare events. Because mixed-effects models only including random intercepts can yield inflated type 1 error rates (Barr, Levy, Scheepers, & Tily, 2013; but see Douglas Bates, Kliegl, Vasishth, & Baayen, 2015; Matuschek, Kliegl, Vasishth, Baayen, & Bates, 2017), we additionally model our results using standard logistic regression (without random effects) while performing Firth bias correction (Firth, 1993), which reduces small-sample bias and yields definite estimates. Firth bias correction was implemented using the brglm2 package (Kosmidis, 2020). In all cases, these models either found the same pattern of statistical results or yielded a larger number of significant fixed effects, suggesting that our random effects models were not inflating type 1 error rates.

*Classification Analyses*

Additionally, in order to facilitate comparison to the results of Study 1, we classified each participant as being sensitive to intentions, outcomes and group membership (non-mutually exclusive) and then compared these percentages across the Punishment and Partner Rejection conditions (see Supplemental Material). We used the same approach as in Study 1, classifying participants as sensitive to a feature (intentions, outcomes and group membership) if, holding the other two features constant, their response differed based upon the levels of that feature (ignoring the direction of that difference).

As in Study 1, we explored the influence of a number of additional variables on this overall pattern of results, especially the interaction between group membership and response type (see Supplemental Material). First, we found that these results were unchanged if insensitive participants were excluded, with the exception that we found additional interactions between group membership, intentions and outcomes, and between response type, intentions and outcomes. Similarly, we continued to find an interaction between response type and group membership when including ingroup identification ratings in our models. However, we now additionally found 2-way interactions between ingroup identification and group membership, outcomes and intentions, as well as a marginal interaction between ingroup identification and response type. Including a predictor for which Decider participants interacted with first yielded no influence of Decider order on the influence of group membership. We continued to find an interaction between response type and group membership, though we found additional interactions between Decider order and intentions and Decider order and outcomes. Including a predictor for attentiveness (as measured by first-try performance on comprehension questions), we found an interaction between attentiveness, outcomes and group membership; attentiveness, response type and outcomes; and between attentiveness, response type and intentions. However, attentiveness did not influence the interaction between response type and group membership, which remained significant even when the role of attentiveness was accounted for. Finally, including CRT performance in our models yielded a marginal interaction between CRT performance, response type and group membership. Inspecting means, this interaction reflected a greater tendency to differentiate outgroup partners from ingroup and neutral partners amongst those higher on CRT performance, relative to those lower on CRT performance. In addition, those higher on CRT performance were less likely to engage in punishment overall and more likely to reject the Decider, relative to those lower on CRT performance. We note, however, that strong conclusions should not be drawn from these results, given their marginal nature. We also found interactions between CRT performance, response type and outcomes and between CRT performance, response type and intentions. However, we continued to find an interaction between response type and group membership when the influence of CRT performance was accounted for. Thus, while these additional factors influenced the role that other factors play in some cases, the interaction between response type and group membership was consistently significant and only ever modulated in the case of the marginal interaction with CRT performance. In total, we consistently found support for the idea that group membership has a strong influence on punishment and has almost no influence on partner choice decisions.

*Participants as the Decider*

When analyzing participants’ decisions when playing as the Decider, responses are binary (Option A versus Option B) and so we model these data using mixed-effects logistic regression, including a random intercept for participants and again taking a model comparison approach.

Finally, as in Study 1, we investigated how participants’ decisions as a Decider were influenced by group membership and whether they might be subject to punishment or partner choice (see Supplemental Material). To summarize, we found that participants were most likely to choose the fair option when the Responder was an ingroup member, slightly less likely to choose the fair option when paired with a neutral individual, and least likely to choose the fair option when paired with an outgroup member, in both response conditions. In Study 2 we no longer found that response type influenced these decisions. We again found that the influence of Responder group membership was stronger when individual differences in ingroup identification were taken into account, such that those highest on ingroup bias were more likely to favor ingroup Responders over outgroup or neutral Responders.

Judgments

We also found no difference in judgment of ingroup and neutral partners (OR = 1.21, SE = 0.11, *z =* 0.69, *p* = 0.49, 95% CI = 0.87–1.34) or neutral and outgroup partners (OR = 1.17, SE = 0.11, *z =* 1.44, *p* = 0.15, 95% CI = 0.95–1.45), though we did find a weak difference in judgment of ingroup and outgroup partners (OR = 1.26, SE = 0.11, *z =* 2.14, *p* = 0.03, 95% CI = 1.02–1.57).
